# Medical Society of the County of Erie

**Published:** 1899-02

**Authors:** F. C. Gram

**Affiliations:** Secretary


### MEDICAL SOCIETY OF THE COUNTY OF ERIE.

REPORTED BY F. C. GRAM, M. D., Secretary.

THE Medical Society of the County of Erie held its annual meeting, January 10, 1899, in the parlors of the Buffalo Academy of Medicine. The President, Dr. LUCIEN HOWE, called the meeting to order at 10.15 a. m., and the secretary read the minutes of the semi-annual and of the two special meetings, which were approved as read.

Dr. WILLIAM WARREN POTTER, chairman of the committee on membership, reported favorably upon the following-named applicants: John Hudson Grant, J. C. Clemesha, Emil Lustig, Abraham L. Weil, Mary Clayton, James W. Charters, N. L. Burnham, Christ A. Weinbach and Charles R. Borzilleri. They were thereupon duly elected to membership.

Applications for membership were received from Drs. Wm. B. May, Charles F. Durand, Montressor Axford and Edward J. Kiepe, which were referred to the committee on membership.

The death of Dr. J. C. GREENE was announced by the president as having occurred on January 3, 1899, and the following memorial was presented by Dr. WILLIAM C. PHELPS, chairman of memorial committee:

### IN MEMORIAM—JOSEPH C. GREENE, M. D.

The members of the Medical Society of the County of Erie acknowledge, in the death of Dr. Joseph C. Greene, the loss of a valued colleague, a worthy citizen and an honored member of society. His life was one of industry, energy, perseverance and integrity. His every act was along these lines. It is said that one of the finest instincts of our nature is that which prompts us to honor the dead. Our deceased brother was worthy of our highest consideration during life, and his death only brings more fully to view his many excellent qualities.

Dr. Greene's life-work was that of a physician. He became a resident of Buffalo in 1863, and has been of us and with us since that year. His great success in his chosen profession was due to a natural aptitude for it, and also to the unusual amount of professional knowledge which he had acquired. He was a most excellent physician, and this society honored him and itself by electing him its president in 1884. He was also at one time president of the fourth district branch of the New York State Medical Association. Dr. Greene was a man of public spirit and a patriot.

In 1885 he was elected to the board of aldermen, and ably represented his ward. He was also an active member, and for a time president, of the Buffalo Historical Society. He took great interest in this institution, which led him to gather, during a trip around the world in 1888 and 1889, a valuable collection of historical articles, which he presented to the society's museum. He was one of the original members and first president of the New York state branch of the Society of Vermonters.

His patriotic impulse caused him to enter with his whole soul into the plan of marking, in some suitable manner, the place of burial of those soldiers of the war of 1812 who died in the military hospital near Williamsville, Erie county; and although under the shadow of a fatal disease, he labored industriously and untiringly, though much beyond his strength, till his object was accomplished.

It will be doing no injustice to the living or the dead members of this society to say that no better specimen of the American general practitioner can be found than was Dr. Joseph C. Greene. In the words of one of our own poets, it may be asked:

> " Why weep ye, then, for him, who having run
> The bound of man's appointed years, at last,
> Life's blessings all enjoyed, life's labors done,
> Serenely to his final rest has passed;
> While the soft memory of his virtues yet
> Lingers, like twilight hues when the bright sun has set?"

*Resolved*, That this memorial be spread on the minutes of this society, and that a copy be sent to the family of the deceased with an expression of our profound sympathy.

The memorial was adopted.

The vice-president, Dr. J. B. COAKLEY, then took the chair and the president read his valedictory address, taking for his subject "Why and how systematic examinations should be made of the eyes of the school children." (See page 495.)

The president requested that the address be discussed, whereupon Henry P. Emerson, superintendent of the public schools of Buffalo, was invited to open the discussion.

Superintendent Emerson said that while the facts were undoubtedly as presented, yet the mental progress of the schools had hitherto demanded so much consideration that proper attention could not be given to the physical. Out of 1,000 school rooms only about thirty had faulty light. These rooms are in old buildings, which the public do not desire torn down at present for economical considerations. The question of towels was another important one. There should either be one towel for each pupil—which for 70,000 pupils would be impracticable—or no towel should be provided at all. Really there is no use for towels in schools, as the children are supposed to wash at home. He desired the opinion of the society on this subject. Since he has held the office of Superintendent, Mr. Emerson said he had tried to obtain adjustable seats, or at least have each room equipped with one row. Pupils are graded according to mental and not physical qualifications. He believed Dr. Howe's suggestion about having teachers make the preliminary examinations of pupils' eyes to be a good one, as parents object to the indiscriminate examination of their children by unauthorised persons. However, there would be no objection to an inspector appointed by the city. There are 1,200 teachers to look after the educational welfare of the 70,000 pupils and he thought there ought to be at least one inspector to look after the physical side. This inspector might feel more free to criticise and suggest if under the supervision of the Health Department than if he were an attache of the School Department.

Dr. Ernest Wende, Health Commissioner of Buffalo, said that the system employed in this city was faulty. The Department of Health is not consulted in the selection of sites or the construction of school buildings; then when irreparable damage was done the department was supposed to remedy it. If consulted the department would never consent to put up a school building next to a factory which annoyed and disturbed the school, as has been done. Last year he advocated the appointment of a medical examiner for schools, but the cry of economy killed it.

Dr. W. C. Callanan thought the entire sanitary condition of the schools should be looked after, as well as the eye-sight of pupils.

Dr. A. A. Hubbell said that the paper was valuable and called attention to a subject variously considered. The suggestions were timely and practicable, but the examiner's duties should not be limited to examining the eye-sight, but include the entire hygienic conditions. He coincided in the views as to the method of examination.

Dr. Hubbell then moved that a committee of three be appointed to examine the entire subject and report at the next meeting.

Dr. Ernest Wende said this subject was not new, but had been considered for five years. He moved in amendment that this com mittee consist of five and that they report at this meeting.

Dr. Hubbell accepted the amendment.

Dr. William Warren Potter said the facts presented were the result of extended observations by Dr. Howe, and discussions of this kind were particularly beneficent to the public, especially when participated in by the Health Commissioner on the one hand, and Superintendent of Education on the other. We have here in this room an object lesson of the difficulties encountered in properly constructing large school buildings with appropriate and scientific regard to light and suitable environment. Each physician should become a public educator in this matter, because favorable public sentiment must be created in order to make the plan practicable.

Dr. B. H. Grove was desirous of having something definite done in this matter. As it was impossible to provide one towel for each pupil, the towels ought to be dispensed with. He also called attention to the overwork and mental crowding of children, as well as to the keeping of children after hours, requiring them to do still more work for some misbehavior.

The motion, as amended, was then adopted, and the president appointed Drs. A. A. Hubbell, Ernest Wende, W. W. Potter, Edward Clark and George F. Cott as such committee. Later they presented the following report, which was adopted :

*Resolved*, That as members of the Medical Society of the County of Erie, we express to the superintendent of education in Buffalo and the school commissioners of Erie county our strong conviction of the advisability of having the teachers in the public schools examine the eyes of all pupils as often as once a year in the manner recommended by the State Board of Health.

*Resolved*, That in a similar manner we express to the mayor, to the boards of aldermen and councilmen, and to the board of supervisors, our sense of the necessity of authorising the health commissioner, as well as the health authorities of the towns, to appoint a physician, who shall be designated as medical inspector of schools, who shall devote his entire attention to the sanitary condition of the schools and the general physical health of the pupils.

(Signed) ALVIN A. HUBBELL,
ERNEST WENDE,
GEO. F. COTT,
WILLIAM WARREN POTTER,
EDWARD CLARK.

Dr. J. B. COAKLEY, chairman of the board of censors, presented the following report :

*To the Medical Society of the County of Erie :*

The board of censors begs to present its semi-annual report as follows :

During the six months last past several cases of alleged illegal practice of medicine have been presented to this board and thoroughly investigated by the combined efforts of the censors and the attorneys employed by this society. In no case during this time has it become necessary to take extreme measures in order to secure an abandonment of the improper practices complained of. In some cases we have been able to bring about by mere suggestion to the practitioner a compliance with the rules and regulations of the medical law, and in other cases by threat of legal proceedings the same result eventually has been accomplished.

This state of affairs goes to show most clearly the good effect resulting from the speedy conviction obtained by our attorneys last spring of an illegal practitioner who had been openly defying the law. This case, as will be remembered, was widely noted in the newspapers, and doubtless has served as a warning to that class of migratory quacks who have been accustomed to touch at Buffalo on their annual round, and, as well, has proved a powerful deterrent to many resident violators of the statute.

We are, therefore, very glad to be able to state that during the past six months the city has been almost entirely free from the class of traveling advertising charlatans who for two or three years previous have been so much in evidence in Buffalo.

We believe that this result has been accomplished largely through the vigorous measures adopted a year ago by this society, and the spreading abroad of the fact that the society was enough in earnest in its campaign against quackery to employ attorneys of its own for the investigation and prosecution of offenders against the medical law.

We might say further that this board and its attorneys have had under advisement the proposition to present to the legislature a bill extending the jurisdiction of the police court of Buffalo, so as to allow the final determination in that court of trials arising under the medical law, as is not now possible.

The large number of prosecutions in New York City is made possible by the speedy trial of all violators of the medical statute before local magistrates, without the necessity of going to the grand jury. If it is thought advisable on all grounds to establish this practice in Buffalo, a suitable bill will be presented at the present session of the legislature.

We may note in this report that the dental society of this city, following the example of this society, has employed local counsel for the prosecution of all offenders against the dental law, and is making a vigorous crusade against quacks in the practice of dentistry.

The number of practising physicians in this city and county is so large, and the number of opportunities for illegal practice so increasingly great, that the board of censors wishes again to call upon the members of the profession throughout the county to report promptly any apparent violations of the law coming to their notice.

The effect of our work during the last two or three years is becoming more and more apparent, and we propose, with the coöperation of the members of the profession, to keep the standard of medical ethics in this county as high as in any part of the state.

Respectfully submitted,

J. B. COAKLEY, Chairman.
THOMAS F. DWYER,
IRVING W. POTTER,
CHARLES E. CONGDON.

The report was accepted and placed on file.

Dr. CHARLES E. CONGDON then read a paper on Some points in the diagnosis of ovarian cysts, which was briefly discussed by Dr. Benedict.

Dr. BENJAMIN F. ROGERS read a paper on Ocular hygiene in the school room, which was discussed by Drs. Abbott and Bayliss. (To be published in the JOURNAL.)

Dr. GEO. F. COTT presented a new atomiser, constructed after plans devised by himself, and explained the mechanism.

Dr. W. C. CALLANAN, librarian, reported that the society's library is at the building of the University of Buffalo, and is used for reference by students and practitioners. Members having medical works which they could spare were asked to contribute.

The report was received and filed.

The treasurer, Dr. EDWARD CLARK, submitted his annual report, which was referred to an auditing committee, consisting of Drs. Benedict, Lytle and Meidenbauer. This committee later reported that they had examined the books and vouchers and found them correct.

The report was received and placed on file.

Dr. W. C. Callanan moved the appointment of a committee on nominations. Adopted.

The chair appointed Drs. Callanan, VanPeyma, W. W. Potter, W. G. Bissell and E. Wende.

The committee presented the following nominations: For president, Dr. J. B. Coakley; for vice-president, Dr. E. H. Ballou, of Gardenville; for secretary, Dr. F. C. Gram; for treasurer, Dr. Edward Clark; for librarian, Dr. W. C. Callanan; for censors, Drs. J. B. Coakley, Irving W. Potter, Thomas F. Dwyer, Charles E. Congdon, F. E. Fronczak.

The report was accepted and on motion of Drs. Callanan and Hoyer the secretary was instructed to cast the ballot of the society for the nominees. This was done and they were thereupon declared elected.

Dr. BENEDICT moved that the authority of the board of censors, to employ counsel as necessity arose, be continued.

Dr. Callanan objected unless the amount be limited to a specific sum. After some discussion the objection was withdrawn and the resolution adopted.

Dr. W. W. Potter moved that the usual honorarium of $50 each be granted the secretary and the treasurer for services during 1898. Adopted.

Dr. Howe then introduced the president-elect, Dr. J. B. Coakley, who assumed the chair, whereupon the meeting was declared adjourned.

